# Variations of the Lingula and Mandibular Ramus in the Context of Sagittal Split Ramus Osteotomy: A Cone Beam Computed Tomography Study Supporting an Ethnic-Centric Approach to Orthognathic Surgery

**DOI:** 10.7759/cureus.67715

**Published:** 2024-08-25

**Authors:** Krishan Sarna, Khushboo Sonigra, Wei Cheong Ngeow, Symon Guthua, Florence Opondo, Hui Wen Tay

**Affiliations:** 1 Department of Oral and Maxillofacial Surgery, Department of Oral Pathology and Oral Medicine, University of Nairobi, Nairobi, KEN; 2 Department of Oral and Maxillofacial Clinical Sciences, Faculty of Dentistry, University of Malaya, Kuala Lumpur, MYS; 3 Department of Oral and Maxillofacial Surgery, Department of Dental and Maxillofacial Radiology, University of Nairobi, Nairobi, KEN

**Keywords:** cone beam computed tomography, mandibular ramus, lingula, orthognathic surgery, sagittal split ramus osteotomy

## Abstract

Objectives: To investigate the ethnic variations concerning the lingula and ramus of the mandible, with particular emphasis on sagittal split ramus osteotomy (SSRO) in orthognathic surgery.

Materials and methods: This study examined Cone beam computed tomography (CBCT) scans from the Kenyan and Malay populations. Lingula morphology was classified into four categories. Morphometric measurements included lingula size, height above the occlusal plane, distance to the second mandibular molar, and distance from its apex to all four mandible borders. Regarding the ramus of the mandible, the thickness of each cortical plate, trabecular bone, and overall thickness were determined at two points. Furthermore, points of fusion of cortical plates were determined in both the vertical and horizontal planes.

Results: Among Kenyans, the triangular shape was most common (46.5%, n = 80 sides), while truncated was most common among Malays (34.4%, n = 57 sides). The overall mean size of lingula differed significantly between Kenyan (7.37 ± 2.19 mm) and Malay (4.14 ± 2.50 mm) populations (p<0.001). The lingula was more located postero-superiorly in Kenyans compared to Malays (p < 0.001). The mean distance from the distal aspect of the second mandibular molar to the lingula was 38.37 ± 4.98 mm among Kenyans, in contrast to 31.95 ± 0.03 mm among Malays (p < 0.001). The Malays exhibited a thicker mandible with a larger trabecular distance (5.99 ± 1.41 mm and 3.41 ± 1.29 mm, respectively) than Kenyans (5.28 ± 1.39 mm and 1.98 ± 0.98 mm, respectively) (p < 0.001). The points of fusion of the cortical plates differed significantly between Kenyans and Malays.

Conclusion: This study focuses on two ethnic groups, Kenyans and Malays, and brings to light the ethnic-based differences in the position of the lingula and the dimensions of the mandibular ramus, both of which are essential considerations in orthognathic surgery. Preoperative consideration of such variations is warranted, potentially mitigating iatrogenic injuries and enhancing successful patient outcomes.

## Introduction

Maxillofacial deformities encompass a spectrum of manifestations, including mandibular prognathism, retrognathism, open bite, or facial asymmetry, culminating in numerous aesthetic and functional challenges significantly impacting the individual's overall quality of life [1]. To address such deformities, orthognathic surgery is pivotal in rectifying skeletal malocclusions [2]. Among the diverse techniques employed, sagittal split ramus osteotomy (SSRO) of the mandible stands out as one of the most common mandibular advancement or setback methods. Despite advantages provided by SSRO, such as the broad area of contact between the two segments and the possibility of precise, rigid fixation, complications such as iatrogenic injury of the inferior alveolar nerve (IAN) and unfavorable fractures have been reported [3].

SSRO involves a strategic sequence of osteotomies involving a horizontal cut on the medial surface of the mandibular ramus, a sagittal osteotomy along the anterior border of the ramus, and a vertical body osteotomy [4]. Of particular concern is the horizontal osteotomy on the medial aspect, which poses a significant risk of iatrogenic injury to the IAN, which traverses the mandibular foramen (MF). To mitigate this risk, a comprehensive understanding of anatomical landmarks on the medial aspect of the ramus is essential for ensuring both the safety and precision of the osteotomy [5]. Notably, the mandibular lingula (ML), situated anterosuperior to the MF, serves as a crucial landmark during this procedure as the position of the osteotomy must be superior to this structure to prevent injury to the IAN. The success of SSRO is also dependent on the correct placement and execution of the sagittal osteotomy, which splits the ramus into a medial and lateral segment. A common complication is an occurrence of unfavorable fractures, commonly referred to as ‘bad splits.’ Such a phenomenon could be attributed to the fusion of the inner and outer cortical plates, resulting in minimal intervening cancellous bone, reducing the ramus's thickness significantly [6,7]. These complications may give rise to mechanical instability and IAN injury while attempting to reposition the segments. Thus, the surgeon’s knowledge regarding these factors is crucial for the safe and effective execution of the SSRO.

The mandibular ramus exhibits substantial variation influenced by the patient’s gender, ethnicity, and geographical location. Notably, variations in the morphology of the ML, morphometry of the ML concerning important surgical landmarks, and the anatomy of the mandibular ramus have been documented to exhibit significant diversity [8]. These parameters are paramount in SSRO, and the knowledge of such variations is crucial for successfully executing the procedure. Cone beam computed tomography (CBCT), a diagnostic tool designed for the maxillofacial region, provides comprehensive diagnostic information. Its accuracy, ability to provide 3-D images, and a tremendously lower radiation dose compared to computed tomography scans make it the imaging modality of choice for investigating such variations [4].

While these measurements could serve as invaluable references in SSRO, a gap in the existing literature pertains to the absence of research within Kenya and Malaysia. Given the pronounced inter-population variations observed, relying on data extrapolated from other populations may lead to inaccuracies, potentially resulting in unfavorable surgical outcomes. Recognizing this critical gap, this study seeks to expand the current understanding by examining the variations of the ML and mandibular ramus in both Kenyan and Malay population groups. Through this analysis, the research establishes population-specific norms and provides valuable insights into the inter-population variations that could influence the success of SSRO. Recognizing these variations during preoperative planning may reduce the risk of iatrogenic injury to critical structures, thereby enhancing patient outcomes.

## Materials and methods

This study received approval from the Kenyatta National Hospital - University of Nairobi Ethics and Research Committee (KNH-UON ERC, protocol number UP757/09/2022) and the Medical Ethics Committee, Faculty of Dentistry, University of Malaya (DF OS2207/0013(P)). To address the objectives of this research, a cross-sectional study was conducted by collecting CBCT scans of patients in a retrospective manner. The study population consisted of CBCT scans of patients aged between 21 and 40 years who were referred to two centers, namely, Maxiray Diagnostic Centre in Nairobi, Kenya, and the Faculty of Dentistry, University of Malaya, Kuala Lumpur, Malaysia, for diagnosis and treatment planning, which included dental implants, maxillofacial surgery, orthodontics, endodontics, and oral pathology. The inclusion criteria of the sample were 1) the absence of any pathology involving the posterior mandibular body, ramus, coronoid process, or condyle of the mandible; 2) CBCT free from distortion, artifacts, or foreign bodies; and 3) the presence of the second molar bilaterally as a landmark for measurement. Any scans that did not meet these criteria or involved maxillofacial growth disorders or an edentulous jaw were excluded from the study. This study was performed in accordance with the Helsinki Declaration of 1975 as revised in 1983.

The equipment used to acquire CBCT scans in both centers was a CS 9300 3D digital imaging system (Carestream Dental LLC, Atlanta, GA). The images were obtained at 90 kV, 10 mA with a voxel size of 76.5 μm^3^, and a 200 μm image resolution. All images were viewed with a 19-inch LCD monitor (HP L1910, Hewlett-Packard Development Co., Palo Alto, CA) with 1280 × 1024 pixel resolution. The 3D Slicer medical imaging platform was used to analyze the scans [9]. To standardize all measurements, the team reviewing the scans (KS, KJS, and HWT) was calibrated on data collection (by FO and WCN). To assess inter-observer (inter-rater) and intra-observer (intra-rater) consistency, 20% of the measurements were re-evaluated and recorded as blinds to the first measurements after two weeks. There were no statistically significant differences between these (p-value >0.05).

**Figure 1:**
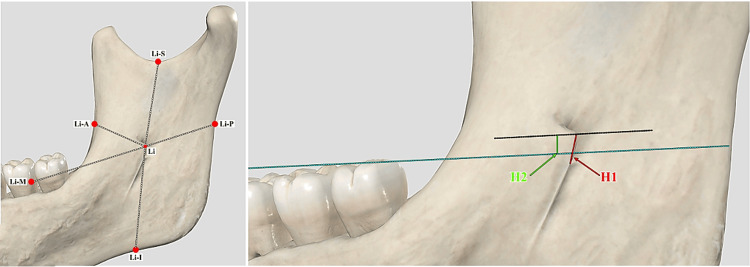
Morphometry of the ML carried out. Li – mandibular lingula (ML); Li-A – distance from ML to the anterior border; Li-P – distance from ML to the posterior border; Li-S – distance from ML to the superior border; Li-I – distance from ML to the inferior border; Li-M – distance from ML to the distal aspect of second mandibular molar; H1 – size of ML; H2 – height of ML above the occlusal plane

The independent variables were ethnicity, gender, and laterality. The ethnicity was categorized as either Kenyan or Malay, gender was either male or female, and laterality was either right or left. The dependent variables were the morphology and morphometry of the ML and the dimensions of the mandibular ramus in both population groups. The morphology of the ML was classified according to Tuli et al., where four distinct categories were present: triangular, truncated, nodular, and assimilated [10]. Regarding the morphometric measurements, the distance of the ML to all four borders of the ramus (anterior, posterior, superior, and inferior) was recorded. Additionally, the distance to the distal aspect of the second mandibular molar (distobuccal cusp tip), the size of the ML, and its height above the occlusal plane (defined using three points - the mesiobuccal cusp of the mandibular first molars and the mid-point of both mandibular central incisors) were also measured. Figure 1 provides a diagrammatic summary of all these measurements.

**Figure 2:**
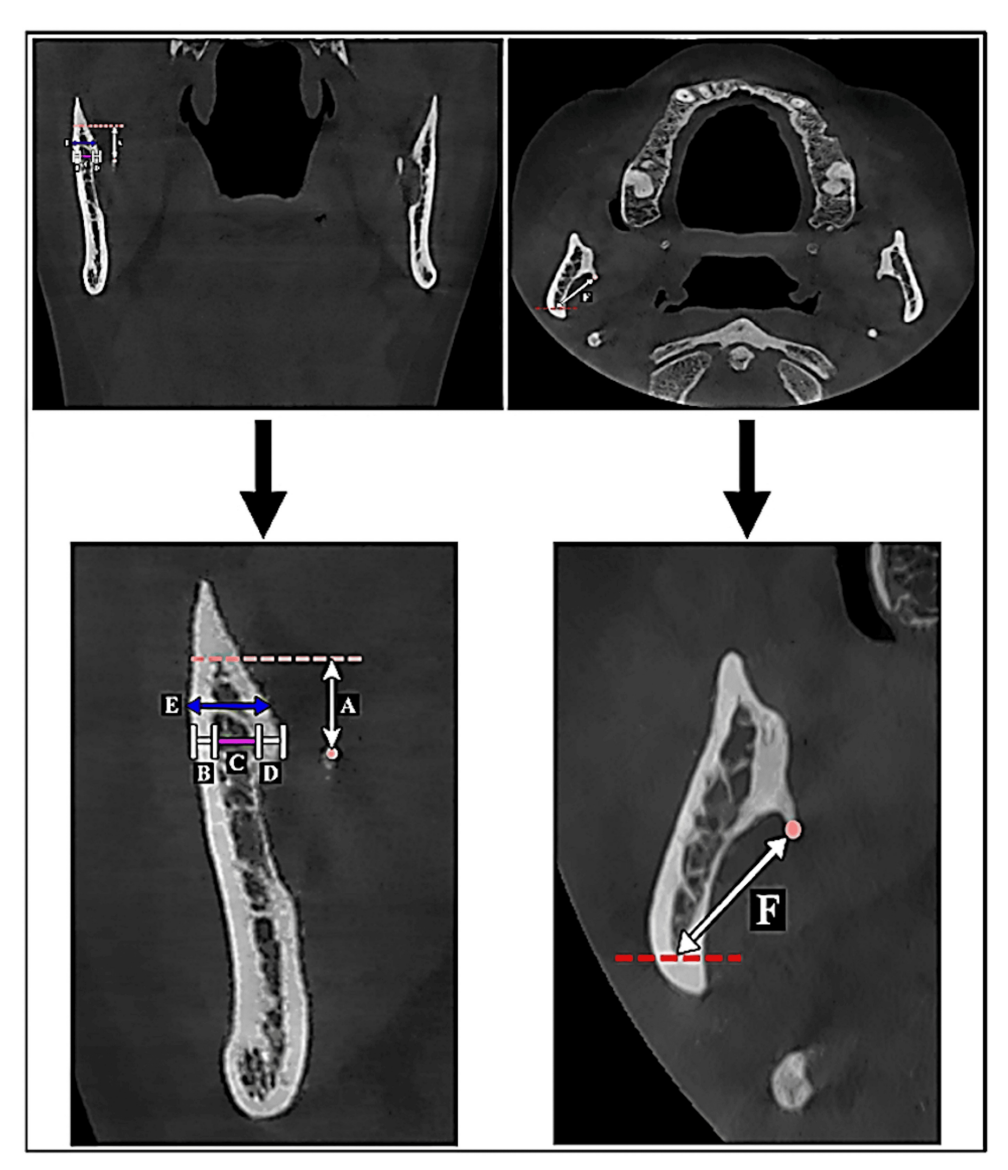
Summary of the measurements of the mandibular ramus taken from the coronal view (top left and bottom left) in the axial view (top right and bottom right). A – vertical distance from the ML to the point of fusion of the buccal and lingual cortical plates; B – buccal cortical thickness; C – thickness of the cancellous bone; D – lingual cortical thickness; E – mandibular thickness of the ramus 4 mm above the ML; F – horizontal distance from the ML to the point of fusion of cortical plates posteriorly

Measurements of the mandibular ramus were performed in two views: coronal and axial. The tip of the ML was used as the reference point for both views. The vertical distance from the ML at which the cortical plates of the ramus fused was recorded. Subsequently, measurement of the mandibular ramus thickness at the level of the ML was performed. The buccal and lingual cortical plate thickness was also measured at the same level. The thickness of the intervening cancellous bone was determined by deducting the sum of the two cortical plates from the overall mandibular thickness. Additionally, the thickness of the ramus was also determined at a level of 4 mm above the ML. In the axial plane, the distance posterior to the ML where the cortical plates fused was recorded (Figure 2).

Statistical analysis was performed using Statistical Product and Service Solutions (SPSS; IBM SPSS Statistics for Windows, Armonk, NY) software. Descriptive statistics were computed and organized in tabular form for each population group. To scrutinize intra-population variations, analysis was conducted to reveal any gender disparities and differences in laterality, which involved using a chi-square test for all categorical variables, while an independent t-test was employed for all continuous variables. Subsequently, a comprehensive inter-population comparison was conducted employing similar tests to reveal differences between the two groups. A threshold of p <0.05 was considered statistically significant.

## Results

The final sample group included data from a total of 338 mandibular rami (169 patients): 172 Kenyan rami (86 patients - 40 males and 46 females) and 166 Malay rami (83 patients - 39 males and 44 females). The mean age of the Kenyans was 31.4 years, while that of the Malays was 30.6 years. Examination of ML morphology within the Kenyan population group revealed that the most prevalent shape was triangularly found in 46.5% (n = 80), followed by the nodular found in 28.5% (n = 49) and truncated (22.1%, n = 38). The assimilated shape was the least commonly found in 2.9% (n = 5). Similar shapes were found bilaterally in 66.4% (n = 57 patients), while unilaterally were found in 33.6% (n = 29 patients). A summary of the specific subtypes has been provided in Table 1. Gender-wise, the triangular and nodular variants were more commonly observed among females, while the truncated and assimilated shapes were seen more among males. Despite the observed differences between gender and ML morphology, the statistical analysis indicated that these were not statistically significant (p > 0.05).

**Table 1:** Comparison of mandibular lingula (ML) morphology with respect to gender among the Kenyan and Malay population groups.

		Triangular % (n sides)	Truncated % (n sides)	Nodular % (n sides)	Assimilated % (n sides)	
Kenyan	Male	22.7 (39)	11.6 (20)	9.9 (17)	2.3 (4)	<0.001
	Female	23.8 (41)	10.5 (18)	18.6 (32)	0.6 (1)	
	Total	46.5 (80)	22.1 (38)	28.5 (49)	2.9 (5)	
Malays	Male	7.8 (13)	17.5 (29)	9.6 (16)	12.0 (20)	
	Female	12.7 (21)	16.9 (28)	13.9 (23)	9.6 (16)	
	Total	20.5 (34)	34.4 (57)	23.5 (39)	21.6 (36)	

Within the Malay population group, the truncated was found in 34.4% (n = 57), and nodular was found in 23.5% (n = 39), followed by assimilated, which was found to be 21.6% (n = 36), while the least common was the triangular shape (20.5%, n = 34). Unilateral variations were common among all morphological types, except for the truncated variant. In terms of gender, a similar pattern to the Kenyan sample was observed, where the truncated and assimilated types were more common among the males. Upon statistical analysis, the differences observed between gender and the ML morphology were not significant (p > 0.05).

An inter-population comparison revealed quite interesting results. The Kenyan population displayed a higher prevalence of triangular and nodular ML shapes, while Malays exhibited a higher occurrence of truncated and assimilated shapes. Notably, Kenyans showed a greater prevalence of bilateral symmetry, whereas Malays displayed a more balanced distribution between bilateral and unilateral variants. Further analysis revealed that these differences in the morphology of the ML between the two population groups were statistically significant (p < 0.001).

The overall mean size of the ML (irrespective of gender or laterality) differed significantly between Kenyan and Malay populations. Kenyans exhibited an average size of 7.37 ± 2.19 mm, whereas Malays showed a statistically significant smaller mean size of 4.14 ± 2.50 mm (p-value < 0.001). Individual, intrapopulation analysis revealed that, among Kenyans exclusively, males exhibited a greater ML size than females, with the right side larger than the left. Conversely, in Malays exclusively, females showed a greater ML size than males, and the left side was larger than the right. Despite these observed trends, these differences within each population were not statistically significant (p > 0.05). However, when analyzing inter-population differences in the size of ML based on gender and laterality, significant differences were observed in each comparison that was made. Table 2 summarizes the size of ML in different ethnicities.

**Table 2:** Size (mean (SD) with 95%CI) and height (mean (SD) with 95%CI) of ML from the occlusal plane in Kenyan and Malays according to gender and side. ML: mandibular lingula

Ethnic Landmark		Side	Kenyan (mm)	Malays (mm)	Inter-population p-value
Size of ML	Overall Average		7.37 ± 2.19	4.14 ± 2.50	<0.001
	Male	Right	8.06 ± 2.59	3.94 ± 2.72	<0.001
		Left	7.33 ± 2.48	3.85 ± 2.44	<0.001
		Total	7.70 ± 2.54	3.89 ± 2.57	<0.001
	Female	Right	7.17 ± 1.99	4.25 ± 2.30	<0.001
		Left	6.92 ± 1.68	4.52 ± 2.41	<0.001
		Total	7.04 ± 1.84	4.39 ± 2.34	<0.001
Height of ML from the occlusal plane	Overall Average		8.06 ± 3.09	8.66 ± 3.48	0.095
	Male	Right	8.30 ± 2.88	9.09 ± 3.35	0.265
		Left	8.52 ± 2.93	9.40 ± 3.6	0.238
		Total	8.41 ± 2.89	9.24 ± 3.46	0.104
	Female	Right	7.95 ± 3.19	7.70 ± 3.23	0.713
		Left	7.48 ± 3.41	8.43 ± 3.73	0.211
		Total	7.71 ± 3.29	8.07 ± 3.49	0.478

The overall height of the ML above the occlusal plane differed slightly, with the Malays exhibiting an increased distance than the Kenyans measuring 8.66 ± 3.48 mm and 8.06 ± 3.09 mm, respectively. Individual, intrapopulation analysis revealed that, among Kenyans exclusively, there were no statistically significant differences with respect to gender or side. However, a notable difference emerged in the Malay group, with males having a significantly greater height above the occlusal plane than the Malay females (p < 0.05). A detailed inter-population comparison of gender and side revealed no statistically significant differences regarding ML height above the occlusal plane (Table 2).

Significant variations in the distance from the distal aspect of the second mandibular molar to the ML were noted between Kenyans and Malays. The mean distance was 38.37 ± 4.98 mm among Kenyans, in contrast to 31.95 ± 3.03 mm among Malays, which was statistically significant (p < 0.001). Evaluation among the Kenyan population exclusively revealed no statistically significant differences with respect to gender or laterality. In contrast, among the Malays, males had a significantly greater distance than females (p < 0.001) (Table 3). Of particular interest, when the two groups were compared based on gender and laterality, significant differences were observed in each comparison made.

**Table 3:** Distance (mean (SD) with 95%CI) of ML to the mandibular second molar in Kenyan and Malays according to gender and side. ML: mandibular lingula

Ethnic Gender	Side	Kenyan (mm)	Malays (mm)	Inter-population p-value
Overall Average		38.37 ± 4.98	31.95 ± 3.03	<0.001
Male	Right	37.31 ± 4.08	32.98 ± 3.39	<0.001
	Left	38.41 ± 4.77	33.29 ± 3.02	<0.001
	Total	37.85 ± 4.41	33.12 ± 3.20	<0.001
Female	Right	38.41 ± 5.46	31.01 ± 2.90	<0.001
	Left	39.34 ± 5.69	30.51 ± 2.83	<0.001
	Total	38.88 ± 5.55	30.77 ± 2.86	<0.001

Regarding morphometric measurements of the ML concerning the borders of the mandibular ramus, the distance to the anterior border was found to be significantly greater in Kenyans (18.82 ± 3.47 mm) in comparison to Malays (17.71 ± 2.44 mm), indicating population-specific differences (Table 4). On the other hand, the distance to the posterior border among Malays (14.56 ± 3.52 mm) was found to be greater than Kenyans (14.25 ± 2.56 mm) (p > 0.05), which shows that the ML was more posteriorly placed among Kenyans than Malays. Furthermore, the distance to the superior border was significantly lower in Kenyans (17.00 ± 3.45 mm) than in Malays (18.14 ± 3.13 mm) (p < 0.05), and the distance to the inferior border was greater among the Kenyans (28.41 ± 4.39 mm) than in Malays (27.79 ± 4.15 mm) (p = 0.183), thus showing that the ML was more superiorly located among the Kenyans. 

**Table 4:** Mean distance of ML to the anterior, posterior, superior, and inferior ramus border in mm. ML: mandibular lingula

	Gender	Kenyan (mm)	Malays (mm)	Inter-population p-value
ML to the anterior border of the ramus (LiA)	Male	18.69 ± 3.87	17.65 ± 2.44	0.012
	Female	18.66 ± 3.10	17.77 ± 1.68	0.017
	Total	18.82 ± 3.47	17.71 ± 2.10	<0.001
ML to the posterior border of the ramus (LiP)	Male	14.42 ± 2.84	15.22 ± 4.28	0.17
	Female	14.33 ± 2.28	13.98 ± 2.53	0.332
	Total	14.25 ± 2.56	14.56 ± 3.52	0.357
ML to the superior border of the ramus (LiS)	Male	17.01 ± 3.09	18.45 ± 3.01	0.003
	Female	16.99 ± 3.76	17.86 ± 3.23	0.097
	Total	17.00 ± 3.45	18.14 ± 3.13	0.002
ML to the inferior border of the ramus (LiI)	Male	28.62 ± 5.24	29.21 ± 4.63	0.454
	Female	28.22 ± 3.50	26.53 ± 3.21	<0.001
	Total	28.41 ± 4.39	27.79 ± 4.15	0.183

A comprehensive analysis of the mandibular ramus revealed notable variations in several measurements when comparing the Kenyan and Malay populations, as highlighted in Table 5. Notably, the mandibular thickness at the level of the ML exhibited a statistically significant difference, with Malays displaying a thicker mandible than Kenyans. It is noteworthy that both gender and laterality played a role in influencing this measurement within both populations. Specifically, females exhibited an increased thickness of the ramus at the level of the ML with a distinct asymmetry observed where the right side was thicker than the left in both populations. The trend observed in ramus thickness at 4 mm above the level of the ML was consistent with that described above.

**Table 5:** Thickness of the ramus (mean (SD) with 95%CI) in Kenyan and Malays according to gender and side.

Ethinic Landmark		Side	Kenyan (mm)	Malays (mm)	Inter-population p-value
Mandible thickness at ML	Overall Average		5.28 ± 1.39	5.99 ± 1.41	<0.001
	Male	Right	5.09 ± 1.38	5.73 ± 1.36	0.041
		Left	4.68 ± 1.12	5.64 ± 1.58	0.003
		Total	4.88 ± 1.27	5.69 ± 1.46	<0.001
	Female	Right	5.87 ± 1.69	6.32 ± 1.39	0.17
		Left	5.50 ± 1.30	6.27 ± 1.32	0.007
		Total	5.68 ± 1.51	6.29 ± 1.35	0.005
p-value (inter-gender)			<0.001	0.007	
Mandible thickness 4 mm above ML	Overall Average		5.28 ± 1.37	6.38 ± 1.37	<0.001
	Male	Right	5.03 ± 1.48	6.15 ± 1.28	<0.001
		Left	5.00 ± 1.40	6.17 ± 1.57	<0.001
		Total	5.01 ± 1.43	6.16 ± 1.42	<0.001
	Female	Right	5.57 ± 1.37	6.74 ± 1.21	<0.001
		Left	5.52 ± 1.27	6.42 ± 1.41	0.002
		Total	5.55 ± 1.31	6.59 ± 1.32	<0.001
p-value (inter-gender)			0.011	0.046	
Buccal cortical thickness	Overall Average		1.93 ± 0.64	1.88 ± 0.63	0.923
	Male	Right	1.91 ± 0.51	1.92 ± 0.47	0.928
		Left	1.81 ± 0.55	1.64 ± 0.79	0.272
		Total	1.86 ± 0.53	1.78 ± 0.66	0.403
	Female	Right	2.06 ± 0.95	2.10 ± 0.68	0.818
		Left	1.92 ± 0.44	1.88 ± 0.50	0.689
		Total	1.99 ± 0.74	1.97 ± 0.60	0.842
p-value (inter-gender)			0.183	0.055	
Lingual cortical thickness	Overall Average		1.38 ± 0.53	0.71 ± 0.65	<0.001
	Male	Right	1.35 ± 0.56	0.66 ± 0.57	<0.001
		Left	1.28 ± 0.49	0.73 ± 0.76	<0.001
		Total	1.31 ± 0.52	0.69 ± 0.67	<0.001
	Female	Right	1.46 ± 0.50	0.63 ± 0.56	<0.001
		Left	1.44 ± 0.56	0.80 ± 0.68	<0.001
		Total	1.45 ± 0.53	0.72 ± 0.62	<0.001
p-value (inter-gender)			0.083	0.766	
Thickness of cancellous bone	Overall Average		1.98 ± 0.98	3.41 ± 1.29	<0.001
	Male	Right	1.83 ± 0.92	3.15 ± 1.17	<0.001
		Left	1.60 ± 0.99	3.27 ± 1.24	<0.001
		Total	1.71 ± 0.96	3.21 ± 1.20	<0.001
	Female	Right	2.35 ± 0.96	3.63 ± 1.34	<0.001
		Left	2.15 ± 1.04	3.59 ± 1.42	<0.001
		Total	2.25 ± 1.00	3.61 ± 1.37	<0.001
p-value (inter-gender)			<0.001	0.047	
The horizontal distance from ML to the point of fusion of buccal and lingual cortical plate	Overall Average		12.48 ± 2.66	11.68 ± 2.30	0.003
	Male	Right	12.01 ± 2.60	11.89 ± 2.18	0.825
		Left	12.40 ± 2.79	12.04 ± 2.36	0.537
		Total	12.20 ± 2.68	11.97 ± 2.26	0.561
	Female	Right	12.38 ± 2.90	11.44 ± 2.38	0.096
		Left	13.07 ± 2.32	11.41 ± 2.28	<0.001
		Total	12.73 ± 2.64	11.43 ± 2.32	<0.001
p-value (inter-gender)			0.194	0.131	
The vertical distance from ML to the point of fusion of buccal and lingual cortical plates	Overall Average		7.50 ± 2.81	8.33 ± 2.69	0.005
	Male	Right	7.05 ± 3.24	7.87 ± 2.61	0.219
		Left	7.49 ± 2.60	8.41 ± 3.06	0.155
		Total	7.27 ± 2.92	8.13 ± 2.83	0.062
	Female	Right	7.75 ± 2.88	8.42 ± 2.51	0.242
		Left	7.66 ± 2.56	8.56 ± 2.65	0.105
		Total	7.70 ± 2.71	8.49 ± 2.57	0.046
p-value (inter-gender)			0.321	0.394	

Regarding the buccal cortex thickness, the right side surpassed that of the left in both populations. However, no statistically significant differences emerged when considering gender and laterality. Conversely, the lingual cortical thickness exhibited noteworthy distinctions between the two groups, with Kenyans demonstrating thicker lingual cortices than Malays. Upon investigating cancellous bone thickness, a pivotal observation emerged, indicating Malays possessed significantly greater thickness than Kenyans. Once again, the right side demonstrated greater thickness than the left, with gender and laterality proving to be statistically significant factors.

The overall average of the horizontal distance from the ML to the point of fusion of the cortical plates displayed statistically significant differences between the Kenyan (12.48 ± 2.66 mm) and Malay (11.68 ± 2.30 mm) populations (p < 0.05), showing that the cortical plates were found to fuse further posteriorly in the horizontal plane among Kenyans. Regarding the vertical distance from the ML to the point of fusion of the cortical plates, Malays were found to exhibit a greater distance (8.33 ± 2.69 mm) than the Kenyans (7.50 ± 2.81 mm) (p < 0.05), thus showing that the cortical plates fused at a shorter distance in the vertical plane among Kenyans.

## Discussion

SSRO stands out as a widely practiced and integral procedure in orthognathic surgery. Despite its widespread acceptance and established nature, this procedure carries inherent risks, notably the potential for iatrogenic injury to the IAN as it enters the MF, along with the risk of undesirable (bad) splits that can precipitate intra-operative and post-operative complications [11]. The success of SSRO is highly dependent upon the precise placement of osteotomies, keeping in mind the vital anatomical structures that lie within the surgical field; thus, a comprehensive understanding of the anatomical variations in the ML and mandibular ramus presents an opportunity to mitigate these complications effectively [12]. The current study, while unable to precisely look into the ML with respect to the mandibular shape of orthognathic patients, still has a role in providing useful information relating to the subject at hand. The challenge in the study, of course, is that the patients attend for dental implants, other maxillofacial surgery, orthodontics, endodontics, and oral pathology, and there is no way that the authors can determine their skeletal pattern without proper SNA points included in the CBCT scan.

According to standard textbooks and illustrations, the shape of the ML is depicted as triangular. This is inaccurate as the ML exhibits significant morphological variants [10]. In the present study, the most common shape found among the Kenyans was triangular, while that among the Malays was truncated. Interestingly, in both groups, the truncated and assimilated variants were more common among males, while the triangular and nodular variants were more common among females. The results reveal significant inter-population differences in the morphology of the ML. Similar to the findings among the Kenyan population, the triangular shape of the ML was also found to be the most commonly observed by Tuli et al. and other researchers within the Indian population [10,13,14]. Additionally, these findings parallel those reported by Lopes et al. and Özalp et al. in the Brazilian and Turkish cohorts, respectively [15,16]. Conversely, in line with the findings in the Malay population, the truncated variant predominated in populations such as the Thai, Southern Indian, and South Africans [17-19]. As a departure from the findings of this study, the Turkish, Japanese, and Indians exhibited the nodular variant as the most prevalent [20-22]. This study revealed an interesting pattern with Malays more frequently exhibiting an assimilated shape of the ML, while Kenyans show a lower occurrence of the same. None of the subjects in both populations show any deviation in the shape of ML that was not described by Tuli et al. [10]. The horizontal osteotomy is typically positioned 'just above the ML,' as outlined in various texts on the subject. However, the elevated frequency of assimilated lingulae (missing lingula) among Malays introduces a heightened challenge in accurately placing the osteotomy.

Investigation into ML size yielded interesting differences, revealing a mean size of 7.37 ± 2.19 mm among Kenyans and 4.15 ± 2.50 mm among Malays. This difference was found to be statistically significant (p < 0.001). Further examination within each population group highlighted gender-specific trends, with Kenyan males displaying a greater ML size than females, while Malay females had a greater ML size than males. Comparing these findings with international literature, similarities were observed between the ML size of Kenyans and Indians (7.41 ± 2.23 mm) [18]. Notably, the size of the ML in the Kenyan population was greater than that found in North Indians (5.5 ± 2.02 mm) and Brazilians (5.82 ± 0.43 mm) but was less than that of the Thai (8.2 ± 2.3 mm), Turkish (7.93 ± 1.75 mm), and Taiwanese (8.07 ± 2.39 mm) [14,17,21,23,24]. Among Malays, the ML size was consistently lower than in all other population groups examined. A smaller ML increases the proximity to the MF, reducing the safety margin [25]. Conversely, a greater ML size provides a buffer zone, reducing the risk of iatrogenic injury to the IAN by increasing the distance of the osteotomy from the foramen [25].

The measurement of height above the occlusal plane is a valuable landmark in guiding osteotomy placement during SSRO, offering an additional avenue to mitigate the risk associated with traversing the MF [10]. Additionally, it is commonly used to predict the location of the MF during IAN blocks on the mandibular teeth in dental procedures [10]. Of note, a majority of studies report the position of the MF as opposed to the ML in relation to the occlusal plane. While such studies are invaluable, the MF is not easily visualized intraoperatively and is, therefore, less likely to be considered a clinical landmark. The ML, on the other hand, can be easily identified. This investigation showed that the height above the occlusal plane was greater in Malays (8.66 ± 3.48 mm) compared to Kenyans (8.06 ± 3.09 mm), with no statistically significant differences between the right and left sides. These findings are like those observed worldwide, with the measurement among the Turkish population being 8.12 ± 2.95 mm [25]. However, the height was found to be greater among the Kenyans and Malays than those reported by Rikhotso et al. among South Africans (average height above the occlusal plane of 6.50 ± 2.42 mm) and Jansisyanont et al. among the Thai (4.50 ± 2.60 mm) [17,19]. This study did not observe cases where the ML was located below the occlusal plane, aligning with findings reported by Rikhotso et al. [19].

Perhaps the best way to determine the position of the ML is in reference to the borders of the mandible, especially intra-operatively, where the surgical field is severely restricted in terms of access and visibility [21]. From the results obtained, the position of the ML among the Kenyans was found to be more posterior and superior to that of the Malays. This is supported by the fact the distance from the anterior border was greater among Kenyans than Malays and the distance between the ML and the superior border was significantly less in Kenyans than in Malays. These results suggest that performing the horizontal osteotomy at a comparable level in Kenyan individuals, as commonly done among Malays, poses a potential risk. This is because, in Kenyans, the ML is positioned more superiorly, increasing the likelihood of the osteotomy intersecting these structures. This highlights the importance of an ethnic-centric approach in SSRO.

In comparison between Kenyans and the global averages on the topic, ML was located more anteriorly among the Indian, Turkish, Brazilian, and Italian groups, while it was located more posteriorly among the Turkish and Korean groups [21,24,26-29]. With respect to Malays, a relatively similar trend is noted. Concerning the distance from the ML to the superior border, the results from this study corroborate findings from other studies across the globe. A summary of all measurements is provided in Table 6.

**Table 6:** Comparison of the height of ML and its distances to the four borders of the mandible against selected populations. ML: mandibular lingula

Author	Population	Size of ML	Li-A	Li-P	Li-S	Li-I	Li-2nd Molar
Jansisyanont et al., 2009 [17]	Thai	8.2 ± 2.3	20.6 ± 2.4	18.0 ± 2.6	16.6 ± 2.9	-	-
Samanta et al., 2012 [14]	North Indian	5.5 ± 2.02	20 ± 2.4	15 ± 2.7	15.4 ± 2.7	-	-
Padmavathi et al., 2014 [18]	India	7.41 ± 2.23	21.32 ± 4.12	19.61 ± 3.33	18.62 ± 3.71	36.05 ± 4.12	34.6 ± 5.14
Rikhotso et al., 2017 [19]	South African	-	20.15 ± 2.85	16.81 ± 2.13	16.32 ± 2.38		33.35 ± 3.6
Sophia et al., 2015 [28]	India	7.4 ± 1.48	17.11 ± 2.32	14.86 ± 2.54	18.71 ± 3.18	30.3 ± 5.11	-
Senel et al., 2015 [27]	Turkish	7.8 ± 2.4	18.5 ± 2.3	16.9 ± 3.5	18.1 ± 3.6	38.3 ± 5.3	-
Monnazzi et al., 2012 [24]	Brazil	5.82 ± 0.43	16.5 ± 2.32	14.63 ± 2.13	16.38 ± 2.59	27.09 ± 5.44	-
Sekerci et al., 2014 [21]	Turkish	7.91 ± 1.74	16.77 ± 2.75	7.78 ± 1.82	23.09 ± 3.67	26.05 ± 3.84	29.45 ± 3.92
Lupi et al., 2021 [26]	Italians	-	16.96 ± 2.40	15.28 ± 2.10	13.87 ± 3.69	31.20 ± 4.35	29.22 ± 3.98
Zhou et al., 2017 [29]	Koreans	-	18.25 ± 2.3	17.6 ± 1.75	15.60 ± 2.5	32.90 ± 3.05	29.55 ± 3.10
Hsu et al., 2020 [23]	Taiwan	8.07 ± 2.39	19.21 ± 3.02	15.22 ± 2.02	20.04 ± 3.16	31.20 ± 3.81	-
Present Study	Kenyan	7.35 ± 2.19	18.82 ± 3.47	14.25 ± 2.56	17.00 ± 3.45	28.41 ± 4.39	38.4 ± 4.98
Present Study	Malay	4.15 ± 2.5	17.71 ± 2.1	14.56 ± 3.52	18.14 ± 3.13	27.79 ± 4.15	31.9 ± 3.03

Measurements of the thickness of the mandibular ramus yielded valuable results in this study. Significant differences were found between the Kenyans and Malays. The thickness of the ramus at the level of the ML and 4 mm above the ML both revealed that the ramus was significantly thinner among the Kenyans in comparison to the Malays. This knowledge is essential during SSRO as it influences the depth of the horizontal osteotomy. The buccal cortical thickness was quite similar between the groups; however, the lingual cortical thickness was significantly higher among the Kenyans than in Malays. This again has a direct implication during horizontal osteotomy as the surgeon can anticipate the thickness, thus regulating the amount of pressure used with the osteotome and anticipating a breach of the cortex [1,4]. The thickness of cancellous bone is a vital parameter in SSRO, as this type of bone is correlated with a predictable pattern of fracture of the mandibular ramus [2]. The thickness of cortical bone was found to be significantly greater in Malays than in Kenyans which may imply that the occurrence of unfavorable splits is higher among Kenyans than Malays. However, further studies are needed to corroborate this hypothesis.

Regarding the level of fusion of the cortical plates, it was noted that the fusion occurred at a higher level among the Malays but at a lower level among the Kenyans. This suggests that the osteotomy placement should not be more than 7 mm above the ML among Kenyans, as this would result in an unpredictable fracture pattern, thus increasing the risk of bad splits and complications during SSRO. In comparison, the fusion of cortical plates occurs at a higher level among the Malays. Comparison of this data to other studies is exceptionally challenging as there is a general dearth of information on the subject. Of the studies that are published, most have focused on the thickness of the ramus in relation to the MF and not the ML. Taking these measurements from the ML may be beneficial as it is a readily identifiable landmark compared to the MF.

The limitation of the present study was governed by the radiographs available. Most CBCT scans were class I with crowding, and of those that were class II or class III, we could not categorize them as such due to the lack of skeletal landmarks required for accurate classification even though the lower jaw may appear longer or shorter. However, future studies are planned that investigate this. The present study establishes important baseline measurements in both population groups and sheds light on the variations of these structures that can aid the surgeon during SSRO.

## Conclusions

SSRO is a widely embraced procedure within oral and maxillofacial surgery, reflecting its pervasive use in clinical practice. However, despite its popularity, the procedure carries inherent risks, notably the potential for iatrogenic injury to the IAN and the risk of undesirable splits. The findings of this study reveal substantial morphological and morphometric variations of the ML and significant variations in the dimensions of the mandibular ramus, both of which are crucial considerations for practitioners undertaking SSRO. The most prevalent shape of ML was triangular among Kenyans and truncated among Malays with significant differences in size. The position of the ML was found to be postero-superior among the Kenyans compared to Malays, which must be considered when placing the medial osteotomy. Furthermore, the mandibular ramus dimensions were also found to vary significantly, being thicker among Malays in comparison to Kenyans. Such differences emphasize the importance of adapting surgical techniques based on population characteristics. Recognizing the ethnic-centric variations highlighted in this research is crucial for minimizing complications and optimizing outcomes in orthognathic surgery.

## References

[1] Park KR, Kim SY, Kim GJ, Park HS, Jung YS (2014). Anatomic study to determine a safe surgical reference point for mandibular ramus osteotomy. J Craniomaxillofac Surg.

[2] Susilo BT, Sulistyani LD, Priaminiarti M, Latief MA (2018). Mandibular ramus thickness based on cone beam computed tomography scan. J Phys Conf Ser.

[3] Noleto JW, Marchiori E, Da Silveira HM (2010). Evaluation of mandibular ramus morphology using computed tomography in patients with mandibular prognathism and retrognathia: relevance to the sagittal split ramus osteotomy. J Oral Maxillofac Surg.

[4] Ittiwhipat P, Prapayasatok S, Tripuwabhrut K, Kiattavorncharoen S, Sriyaranya N (2021). Comparison of lingual cortical thickness, mandibular ramus thickness, and pattern of cancellous bone distribution at the mandibular ramus between skeletal class I and class III in Thai samples. J Oral Maxillofac Surg Med Pathol.

[5] Park CS, Park JK, Kim H, Han SS, Jeong HG, Park H (2012). Comparison of conventional lateral cephalograms with corresponding CBCT radiographs. Imaging Sci Dent.

[6] Perez D, Ellis E 3rd (2020). Complications of mandibular fracture repair and secondary reconstruction. Semin Plast Surg.

[7] Ribeiro DP, Gandelmann IH, Medeiros PJ (2006). Comparison of mandibular rami width in patients with prognathism and retrognathia. J Oral Maxillofac Surg.

[8] Lascala CA, Panella J, Marques MM (2004). Analysis of the accuracy of linear measurements obtained by cone beam computed tomography (CBCT-NewTom). Dentomaxillofac Radiol.

[9] (2024). 3D Slicer image computing platform. https://www.slicer.org.

[10] Tuli A, Choudhry R, Choudhry S, Raheja S, Agarwal S (2000). Variation in shape of the lingula in the adult human mandible. J Anat.

[11] Leung YY, Wang R, Wong NS, Li DT, Au SW, Choi WS, Su YX (2021). Surgical morbidities of sagittal split ramus osteotomy versus intraoral vertical ramus osteotomy for the correction of mandibular prognathism: a randomized clinical trial. Int J Oral Maxillofac Surg.

[12] Kim HJ, Lee HY, Chung IH, Cha IH, Yi CK (1997). Mandibular anatomy related to sagittal split ramus osteotomy in Koreans. Yonsei Med J.

[13] Gupta S, Pandey K (2014). Morphological analysis of the lingula in dry mandibles of individuals in North India. IOSR J Dent Med Sci.

[14] Samanta P, Kharb P (2012). Morphological analysis of the lingula in dry adult human mandibles of North Indian population. J Cranio-Maxillary Dis.

[15] Özalp Ö, Salım Arı H, Bilgin B (2020). Morphologic and morphometric analysis of mandibular lingula. Clin Anat.

[16] Lopes PT, Pereira GA, Santos AM (2010). Morphological analysis of the lingula in dry mandibles of individuals in Southern Brazil. J Morphol Sci.

[17] Jansisyanont P, Apinhasmit W, Chompoopong S (2009). Shape, height, and location of the lingula for sagittal ramus osteotomy in Thais. Clin Anat.

[18] Padmavathi G, Varalakshmi K, Tiwari S, Roopashree K (2014). A morphological and morphometric study of the lingula in dry adult human mandibles of South Indian origin and its clinical significance. Int J Health Sci Res.

[19] Rikhotso R, Munsamy C (2017). A morphological study of the lingula in South Africans in relation to sagittal split osteotomy. S Afr Dent J.

[20] Ahn BS, Oh SH, Heo CK, Kim GT, Choi YS, Hwang EH (2020). Cone-beam computed tomography of mandibular foramen and lingula for mandibular anesthesia. Imaging Sci Dent.

[21] Sekerci AE, Sisman Y (2014). Cone-beam computed tomography analysis of the shape, height, and location of the mandibular lingula. Surg Radiol Anat.

[22] Varma CL, Sameer PA (2013). Morphological variations of lingula in South Indian mandibles. Res Rev J Med Health Sci.

[23] Hsu KJ, Tseng YC, Liang SW, Hsiao SY, Chen CM (2020). Dimension and location of the mandibular lingula: comparisons of gender and skeletal patterns using cone-beam computed tomography. Biomed Res Int.

[24] Monnazzi MS, Passeri LA, Gabrielli MF, Bolini PD, de Carvalho WR, da Costa Machado H (2012). Anatomic study of the mandibular foramen, lingula and antilingula in dry mandibles, and its statistical relationship between the true lingula and the antilingula. Int J Oral Maxillofac Surg.

[25] Akcay H, Kalabalık F, Tatar B, Ulu M (2019). Location of the mandibular lingula: comparison of skeletal class I and class III patients in relation to ramus osteotomy using cone-beam computed tomography. J Stomatol Oral Maxillofac Surg.

[26] Lupi SM, Landini J, Olivieri G, Todaro C, Scribante A, Rodriguez Y Baena R (2021). Correlation between the mandibular lingula position and some anatomical landmarks in cone beam CT. Healthcare (Basel).

[27] Senel B, Ozkan A, Altug HA (2015). Morphological evaluation of the mandibular lingula using cone-beam computed tomography. Folia Morphol (Warsz).

[28] Mahima Sophia M, Anupriya A, Kalpana R (2015). A morphometric and morphological study of mandibular lingula and its clinical significance. Int J Med Res Rev.

[29] Zhou C, Jeon TH, Jun SH, Kwon JJ (2017). Evaluation of mandibular lingula and foramen location using 3-dimensional mandible models reconstructed by cone-beam computed tomography. Maxillofac Plast Reconstr Surg.

